# Type I Interferon Receptor Deficiency in Dendritic Cells Facilitates Systemic Murine Norovirus Persistence Despite Enhanced Adaptive Immunity

**DOI:** 10.1371/journal.ppat.1005684

**Published:** 2016-06-21

**Authors:** Timothy J. Nice, Lisa C. Osborne, Vesselin T. Tomov, David Artis, E. John Wherry, Herbert W. Virgin

**Affiliations:** 1 Department of Molecular Microbiology and Immunology, Oregon Health and Science University, Portland, Oregon, United States of America; 2 Department of Microbiology and Immunology, University of British Columbia, Vancouver, British Columbia, Canada; 3 Department of Medicine, Division of Gastroenterology, University of Pennsylvania Perelman School of Medicine, Philadelphia, Pennsylvania, United States of America; 4 Department of Microbiology and Immunology, and Joan and Sanford I. Weill Department of Medicine, Weill Cornell Medical College, New York, New York, United States of America; 5 Department of Microbiology, and Institute for Immunology, University of Pennsylvania Perelman School of Medicine, Philadelphia, Pennsylvania, United States of America; 6 Department of Pathology and Immunology, Washington University School of Medicine, St. Louis, Missouri, United States of America; University of Pittsburgh, UNITED STATES

## Abstract

In order for a virus to persist, there must be a balance between viral replication and immune clearance. It is commonly believed that adaptive immunity drives clearance of viral infections and, thus, dysfunction or viral evasion of adaptive immunity is required for a virus to persist. Type I interferons (IFNs) play pleiotropic roles in the antiviral response, including through innate control of viral replication. Murine norovirus (MNoV) replicates in dendritic cells (DCs) and type I IFN signaling in DCs is important for early control of MNoV replication. We show here that the non-persistent MNoV strain CW3 persists systemically when CD11c positive DCs are unable to respond to type I IFN. Persistence in this setting is associated with increased early viral titers, maintenance of DC numbers, increased expression of DC activation markers and an increase in CD8 T cell and antibody responses. Furthermore, CD8 T cell function is maintained during the persistent phase of infection and adaptive immune cells from persistently infected mice are functional when transferred to *Rag1* ^*-/-*^ recipients. Finally, increased early replication and persistence are also observed in mixed bone marrow chimeras where only half of the CD11c positive DCs are unable to respond to type I IFN. These findings demonstrate that increased early viral replication due to a cell-intrinsic innate immune deficiency is sufficient for persistence and a functional adaptive immune response is not sufficient for viral clearance.

## Author Summary

In vertebrates, innate immunity provides early control of viral infection and initiates adaptive immunity. It is generally thought that a virus must either avoid or evade an adaptive immune response to persist within a vertebrate host. The role of innate immunity in preventing viral persistence, apart from its role in initiating an adaptive response, is not completely understood. Type I interferon (IFN) is a cytokine with pleiotropic roles in the antiviral response, including innate control of viral replication. Murine norovirus (MNoV) replicates in cells of the innate immune system, including dendritic cells (DCs), and IFN signaling in DCs is important for early control of MNoV replication. We found here that MNoV persists when DCs are unable to respond to type I IFN. MNoV persistence is correlated with increased adaptive immune responses, likely reflective of increased early viral replication. MNoV also persists in chimeric mice with a 50% mix of wild-type and type I IFN receptor deficient DCs, consistent with a cell-intrinsic role for IFN responses in preventing MNoV persistence. These findings demonstrate that an innate immune deficiency can result in viral persistence apart from its role in generating an adaptive immune response.

## Introduction

The immune response to many commonly encountered viral infections results in viral clearance. Therefore, continuously replicating viral infections represent scenarios of an ineffective immune response or immune tolerance. Mechanistic studies of persistently replicating viral infections including lymphocytic choriomenengitis virus (LCMV) and murine hepatitis virus (MHV) mouse models have provided numerous insights into immune mechanisms of viral persistence [1–4]. In general, study of these and other models have focused on adaptive immune tolerance or the loss of adaptive immune function in determining viral persistence [5–8]. Thus, a paradigm has emerged that viral persistence is linked to defective or tolerant adaptive immune responses [3].

Noroviruses (NoVs) are a leading cause of epidemic viral gastroenteritis worldwide. Human NoV and the closely related murine NoV (MNoV) also establish persistent asymptomatic infection, which may contribute to spread and population-level persistence in between outbreaks [9–12]. Study of MNoV strains that differ in persistence has begun to identify viral and host correlates of persistent infection. MNoV strain CW3 is cleared whereas strain CR6 persists in wild type mice [10,13]. We recently discovered that persistent intestinal CR6 infection is cleared by IFN-λ-stimulated innate responses with no requirement for an adaptive immune response [14,15]. IFN-λ is closely related to type I IFN, but with more specialized roles at epithelial surfaces [16]. This finding suggests that the adaptive immune response is not always necessary for control of persistent infection and that IFN responses play a role in MNoV persistence.

Whereas IFN-λ clears intestinal CR6 persistence, the type I IFN response prevents systemic spread of persistent infection [15]. CW3 spreads systemically and is controlled early by the type I IFN response [13,17]. Therefore, in the present study, we sought to identify the role of type I IFN in control of systemic CW3 persistence. We found that type I IFN receptor (IFNAR) expression on dendritic cells (DCs) was necessary for CW3 clearance. The adaptive immune response generated in mice with IFNAR deficient DCs was of increased magnitude, commensurate with the increased early viral replication, and the functional capacity of adaptive immune cells was maintained during persistent infection. Therefore, CW3 persistence in this model represents an example of viral persistence due to innate rather than adaptive immune deficiency.

## Results

### Type I IFN response in myeloid cells prevents viral persistence

**Fig 1:**
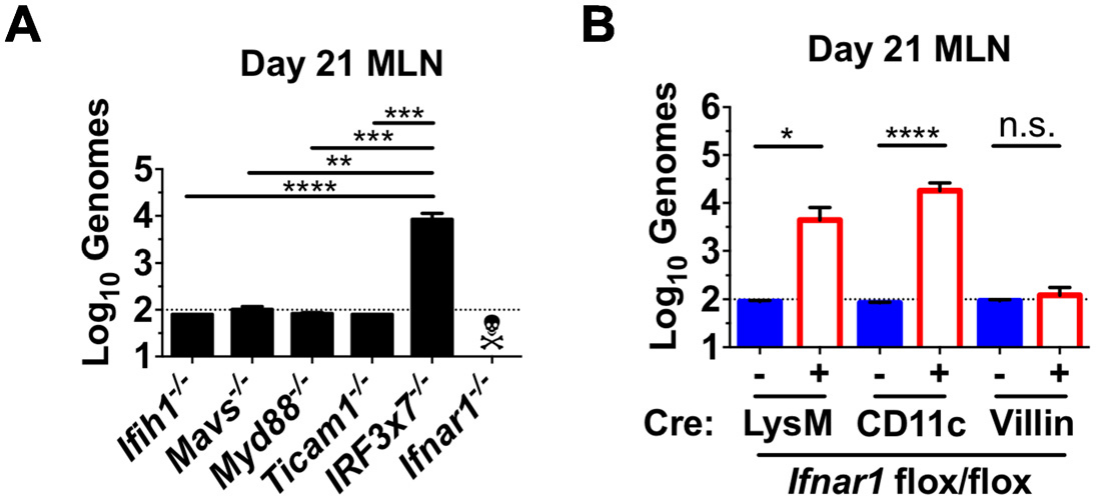
Type I IFN response in myeloid cells prevents viral persistence. *Ifih1* ^*-/-*^, *Mavs* ^*-/-*^, *Myd88* ^*-/-*^, *Ticam1* ^*-/-*^, *IRF3x7* ^*-/-*^, and *Ifnar1* ^*-/-*^ mice (**A**) or Cre-positive and Cre-negative littermates from *Ifnar1* ^*flox/flox*^ x LysM-cre, *Ifnar1* ^*flox/flox*^ x CD11c-cre, and *Ifnar1* ^*flox/flox*^ x villin-cre crosses (**B**) were tested for the ability to clear CW3 infection. Mice were inoculated with CW3, and viral genomes were quantified in the MLN on day 21. Data is combined from at least two experiments with a total of 5–11 mice per group. Statistical significance determined by Kruskal-Wallis test. n.s = p>0.05, * = p≤0.05, ** = p≤0.01, *** = p≤0.001, **** = p≤0.0001.

Cytosolic RNA receptors and membrane toll-like receptors cooperate to sense viral infection and lead to induction of type I and type III IFNs. We examined the ability of CW3 to persist in mesenteric lymph nodes (MLN) of mice lacking the cytosolic RNA sensor MDA5 (*Ifih1* ^*-/-*^) or the downstream signal transducing molecule MAVS (*Mavs* ^*-/-*^). We also examined mice lacking signal transducers for toll like receptor (TLR) signaling Myd88 or Trif (*Myd88* ^*-/-*^ or *Ticam1* ^*-/-*^). None of these individual signaling molecules was critical for CW3 clearance (Fig 1A). However, mice lacking the transcription factors IRF3 and IRF7 (*Irf3x7* ^*-/-*^), which are utilized for interferon gene transcription by both cytosolic sensing and TLR pathways, failed to clear CW3 (Fig 1A). Thus, global defects in induction of IFN lead to viral persistence.

*Ifnar1* ^*-/-*^ mice succumb to CW3 infection within seven days indicating that type I IFN responses are critical for control of CW3 (Fig 1A)[17]. It was recently shown that mice with selective deletion of *Ifnar1* in macrophages (*Ifnar1* ^*flox/flox*^ x LysM-cre) or DCs (*Ifnar1* ^*flox/flox*^ x CD11c-cre) also exhibit increased CW3 titers early after inoculation [17]. However, unlike *Ifnar1* ^*-/-*^ mice, these lineage-specific *Ifnar1* deficient mice survive CW3 infection. To assess viral clearance in these mice, we sacrificed mice 21 days after inoculation and measured viral genomes in the MLN. Persistent CW3 was detected in mice with either LysM-specific or CD11c-specific *Ifnar1* deficiency (Fig 1B). In contrast, cre-negative littermate control mice or mice lacking IFNAR on Villin-expressing intestinal epithelial cells (*Ifnar1* ^*flox/flox*^ x Villin-cre) were able to clear CW3 infection (Fig 1B). Together, these data indicate that reduced induction of IFN or failure of myeloid cell subsets to respond to type I IFN results in CW3 persistence.

### Type I IFN response in myeloid cells prevents systemic persistence

**Fig 2:**
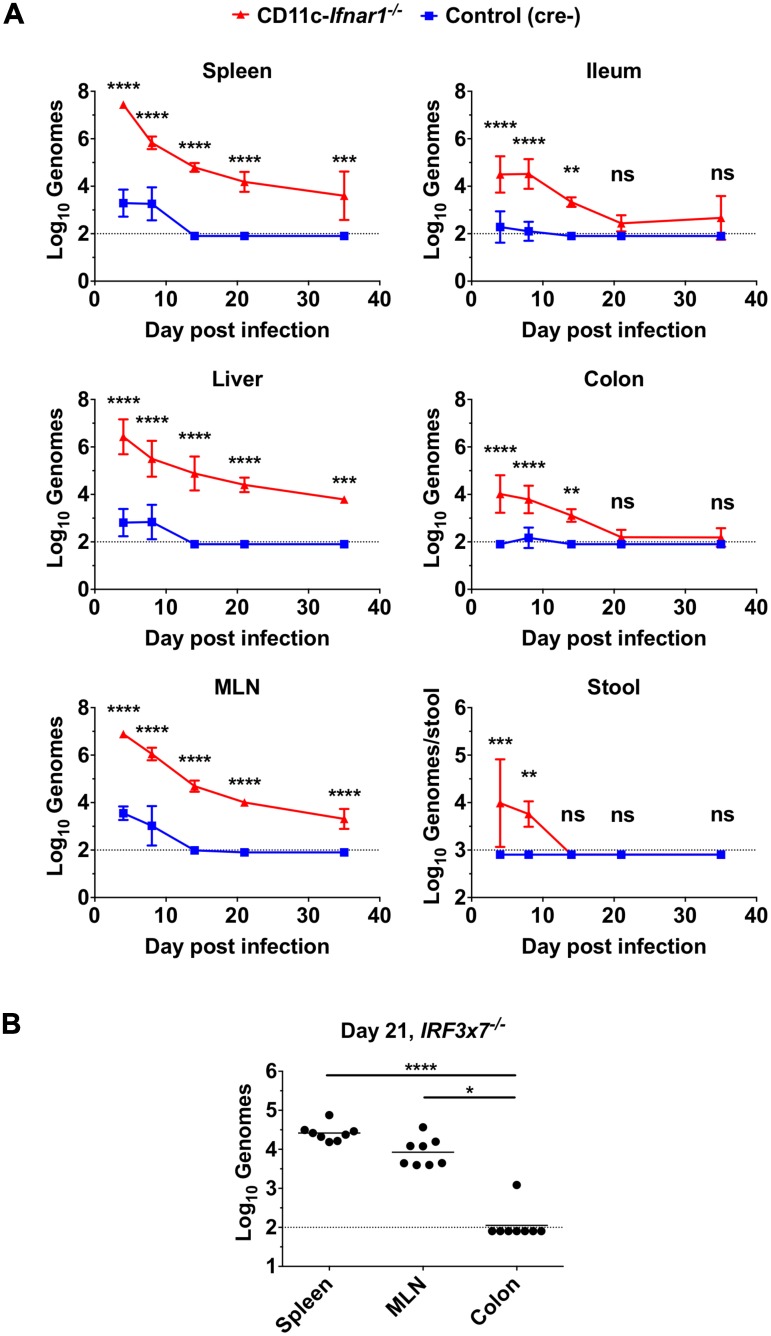
Type I IFN response in myeloid cells prevents systemic persistence. CD11c-*Ifnar1* ^-/-^ mice and littermate controls (**A**) or *IRF3x7* ^*-/-*^ mice (**B**) were inoculated with CW3 and the indicated tissues were collected on days four, eight, 14, 21, and 35 for viral genome quantification by qPCR. Tissues from *IRF3x7* ^*-/-*^ mice (**B**) were collected on day 21 only. Data is combined from at least two experiments with a total of three to eight mice per group. Statistical significance was determined by 2-way ANOVA (A) or Kruskal-Wallis test (B). n.s = p>0.05, * = p≤0.05, ** = p≤0.01, *** = p≤0.001, **** = p≤0.0001.

In immunocompetent mice, the persistence of MNoV strain CR6 is specifically localized to the intestine and MLN whereas CW3 spreads systemically but does not persist [13,15]. Therefore, we assessed the tropism of CW3 over time in mice with CD11c-specific deletion of *Ifnar1* (subsequently referred to as CD11c-*Ifnar1* ^-/-^). Viral genome copies were dramatically increased in CD11c-*Ifnar1* ^-/-^ mice four days after inoculation in systemic and intestinal tissues as well as in the stool (Fig 2A). The greatest differences in genome copy number on day four were in the spleen (15,000-fold), liver (4,000-fold) and MLN (2,000-fold). CW3 was cleared from all tissues in control mice by 14 days after inoculation. The high titers of CW3 in CD11c-*Ifnar1* ^-/-^ mice were partly controlled between day 4 and day 14, with reductions in viral genomes between 8–400 fold (Fig 2A). However, persistence of viral genomes was observed in the spleen, liver and MLN of CD11c-*Ifnar1* ^-/-^ mice for at least 35 days (Fig 2A). CW3 was only sporadically detected in the ileum and colon of CD11c-*Ifnar1* ^-/-^ mice after day 21 and was undetectable in the stool of CD11c-*Ifnar1* ^-/-^ mice after day 14 (Fig 2A). Thus, CW3 persistence in CD11c-*Ifnar1* ^-/-^ was primarily localized to systemic rather than intestinal tissues. Similarly, CW3 persisted in the spleen and MLN but not in the colon of *Irf3x7* ^*-/-*^ mice (Fig 2B). These data are consistent with a primary role of the type I IFN response in DCs broadly limiting early viral replication and preventing viral persistence in systemic tissues.

**Fig 3:**
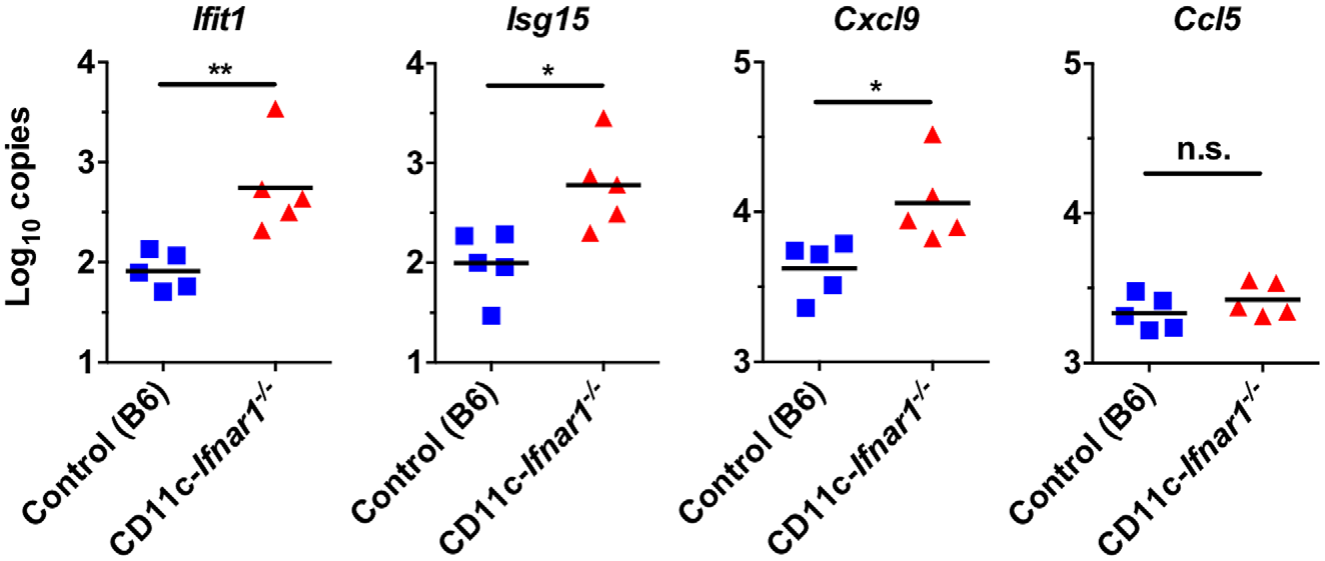
Increased type I IFN stimulated genes in in CD11c-*Ifnar1* ^-/-^ mice. CD11c-*Ifnar1* ^-/-^ mice and C57BL/6 controls were inoculated with CW3 and spleens were collected on days three for quantification of the indicated interferon stimulated genes by qPCR. Data is combined from two experiments. Statistical significance was determined by unpaired t test. n.s = p>0.05, * = p≤0.05, ** = p≤0.01.

The early increase in viral genomes by >1000-fold in systemic tissues suggested that interferon-stimulated genes (ISGs) and other genes related to IFN signaling or recruitment of immune cells may not be appropriately regulated in CD11c-*Ifnar* ^*-/-*^ mice. Therefore, we quantified representative ISGs and chemokines in the spleen on day 3 after inoculation to determine if there was a broad defect in induction of the anti-viral transcriptional response. The cell-intrinsic antiviral mediators *Ifit1* and *Isg15* were present in significantly greater quantities in CD11c-*Ifnar1* ^-/-^ mice (Fig 3). Additionally, transcripts for the T cell chemokine CXCL9 were increased and transcripts for the macrophage chemokine CCL5 were unchanged in CD11c-*Ifnar1* ^-/-^ mice (Fig 3). These data demonstrate that IFN-stimulated genes and chemokines are similar or increased in CD11c-*Ifnar1* ^-/-^ mice.

### DCs have increased activation markers in CD11c-*Ifnar1* ^-/-^ mice

**Fig 4:**
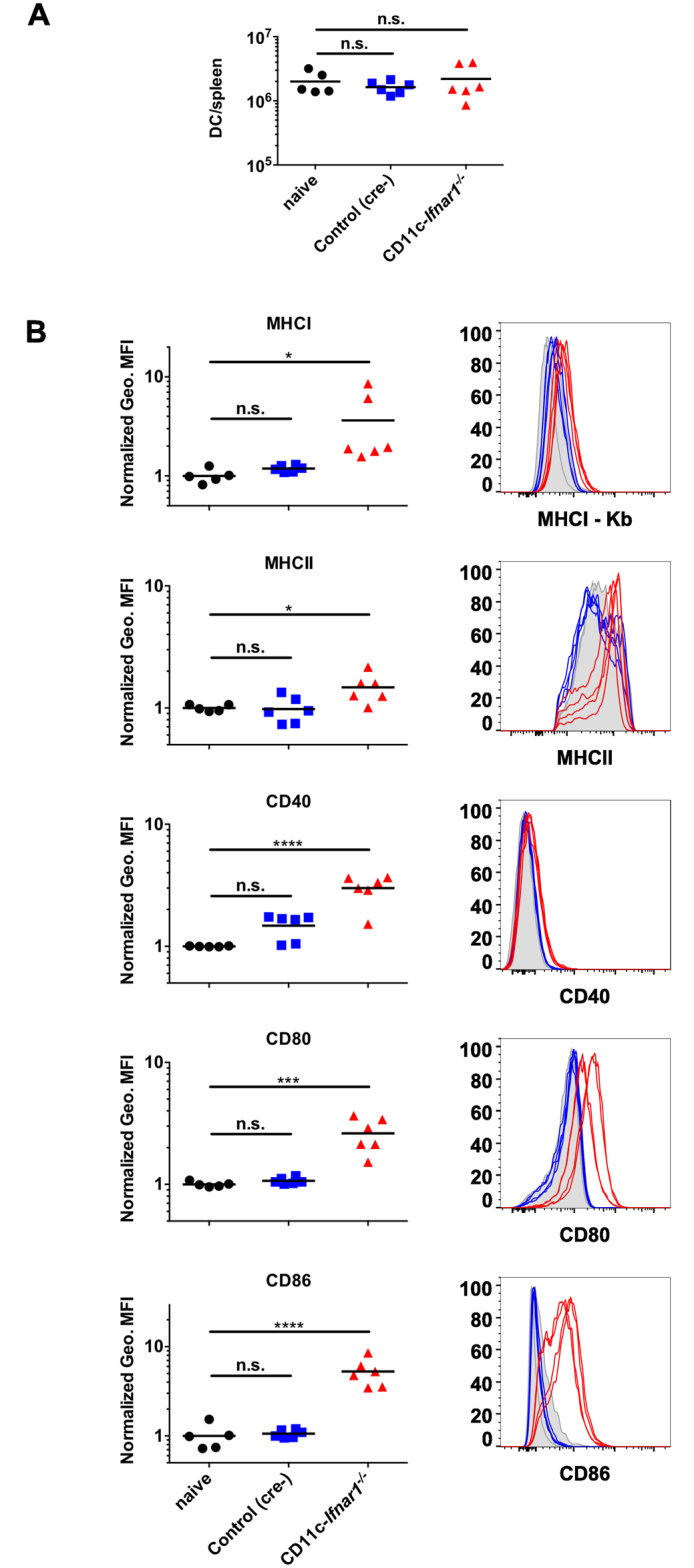
DCs have increased activation markers in CD11c-*Ifnar1* ^-/-^ mice. DCs (CD3-negative, CD19-negative, CD11c-positive, MHCII-positive) from CD11c-*Ifnar1* ^-/-^ mice and littermate controls (cre-negative) 3 days after innoculation with CW3 were quantified (**A**) and stained for surface markers (**B**). The geometric mean fluorescence intensity (Geo. MFI) is shown for MHC molecules MHCI-K^b^ and MHCII-IA/IE, and markers of activation: CD40, CD80, and CD86. Geometric MFIs were normalized in each experiment to the average geometric MFI of each marker on DCs from naïve mice (uninfected cre-positive and cre-negative littermates). Representative histograms are shown. Data is combined from two experiments and individual mice are represented by each data point. Statistical significance was determined by 1-way ANOVA. n.s = p>0.05, * = p≤0.05, *** = p≤0.001, **** = p≤0.0001.

CW3 persists in *Rag1* ^-/-^ mice [18], which indicates that the absence of an adaptive immune response permits CW3 persistence. DC activation and antigen presentation is central to initiation of the adaptive immune response, and CW3 replicates in myeloid cells, including DCs [19]. Therefore, we tested whether the increased viral replication in CD11c-*Ifnar1* ^-/-^ mice correlated with changes in DC number or activation. Three days after inoculation, the number of DCs in CD11c-*Ifnar1* ^-/-^ mice was unchanged relative to naïve mice or cre-negative littermate controls (Fig 4A). Furthermore, DCs in CD11c-*Ifnar1* ^-/-^ mice exhibited increased cell surface expression of MHCI (K^b^), MHCII, and costimulatory molecules CD40, CD80, and CD86 compared to littermate controls (Fig 4B). These data demonstrate that type I IFN signaling on DCs is not necessary for their maturation in this context. In fact, we observe increased activation of DCs in CD11c-*Ifnar1* ^-/-^ mice correlating with the increase in viral replication (Figs 1B and 2A).

NK cells express low levels of CD11c and, therefore, may have altered expression of IFNAR1 in CD11c-*Ifnar1* ^-/-^ mice. Indeed, when we stained NK cells from the spleen three days after inoculation with CW3, expression of CD11c was similar and expression of IFNAR1 was modestly reduced in CD11c-*Ifnar1* ^-/-^ mice. We further assessed NK cell numbers and expression of the surface markers KLRG1, CD62L, and Ly6C to determine if NK cell recruitment or differentiation was altered by the reduced IFNAR1 expression. NK cell numbers were unchanged (S1A Fig); NK cell expression of KLRG1 and Ly6C was not changed and expression of CD62L was modestly increased in CD11c-*Ifnar1* ^-/-^ mice (S1B Fig). These data suggest that a modest reduction in IFNAR1 expression on NK cells does not alter their recruitment or substantially change their activation following CW3 infection.

### CW3 persistence in CD11c-*Ifnar1* ^-/-^ is associated with enhanced humoral immunity

**Fig 5:**
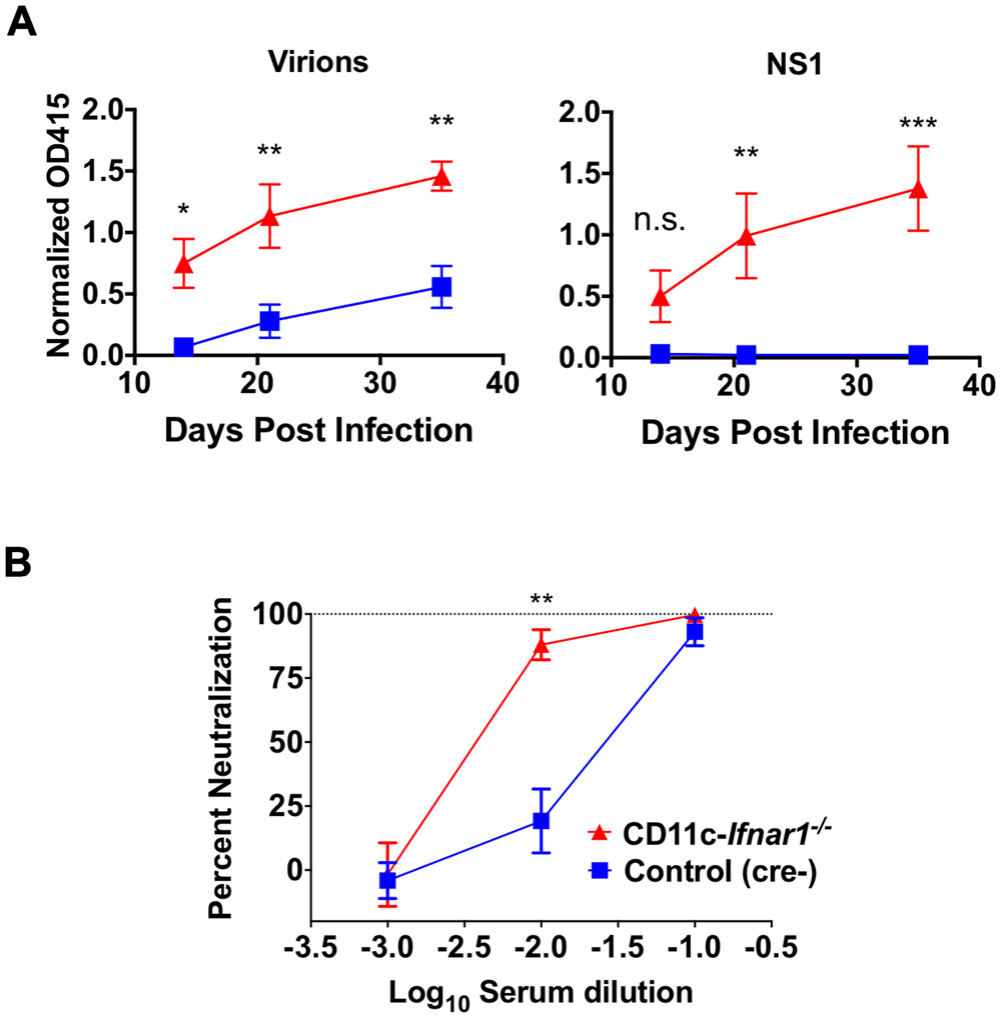
CW3 persistence in CD11c-*Ifnar1* ^-/-^ mice is associated with enhanced humoral immunity. Serum was collected from CD11c-*Ifnar1* ^-/-^ mice and littermate controls 14, 21, and 35 days after infection with CW3. Binding of serum IgG to plate-bound virons or NS1 protein was determined by ELISA (**A**). Dilutions of day 35 serum were incubated with CW3 and the neutralization of viral infection was determined by plaque assay (**B**). Data is combined from two experiments with a total of five to eight mice per data point. Statistical significance was determined by 2-way ANOVA. n.s = p>0.05, * = p≤0.05, ** = p≤0.01, *** = p≤0.001.

Antibody responses are important for keeping persistent viral infections in check [20], making loss of humoral immunity in CD11c-*Ifnar1* ^-/-^ mice a potential basis for viral persistence. To assess the humoral immune response to CW3 in CD11c-*Ifnar1* ^-/-^ mice, we measured the antibody response to viral particles or the NS1 protein by ELISA. Titers of serum antibodies to both viral particles and the viral protein NS1 were significantly higher (3- and 70-fold respectively) in CD11c-*Ifnar1* ^-/-^ mice compared to littermate controls (Fig 5A). To determine if the increased antibody titers resulted in enhanced neutralization of viral particles, we performed a serum neutralization assay. Serum from CD11c-*Ifnar1* ^-/-^ mice was more effective at neutralization of PFUs than serum from littermate controls at the 10^−2^ dilution (Fig 5B). Together, these data demonstrate that there is increased seroconversion and virus neutralization in CD11c-*Ifnar1* ^-/-^ mice. Thus, persistence of CW3 in these mice is not correlated with defective humoral immunity, and instead the humoral immune response in persistently infected CD11c-*Ifnar1* ^-/-^ mice is enhanced.

### CW3 persistence in CD11c-*Ifnar1* ^-/-^ mice is associated with enhanced CD8 T cell expansion

**Fig 6:**
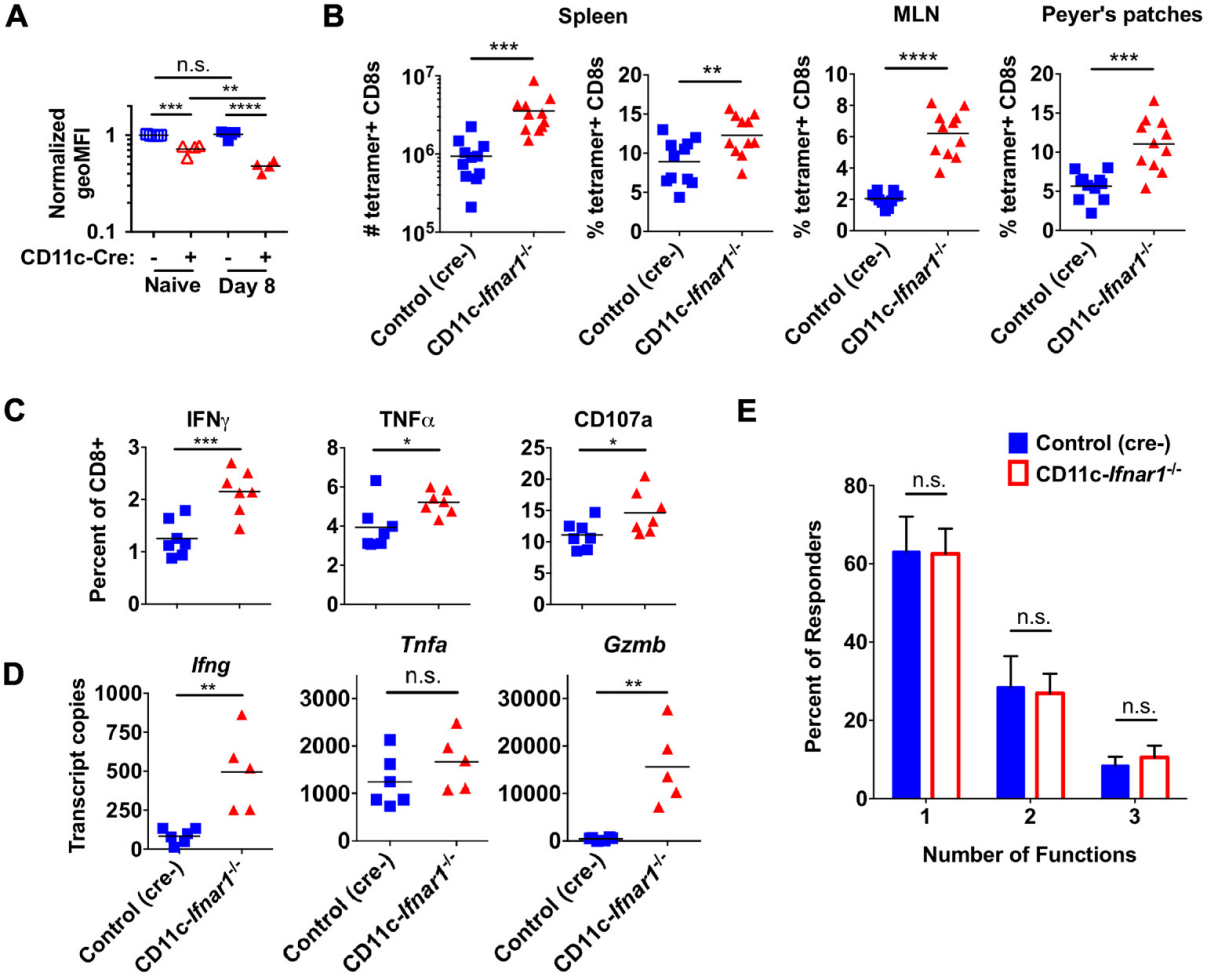
CW3 persistence in CD11c-*Ifnar1* ^-/-^ mice is associated with enhanced CD8 T cell expansion. Cells were collected from spleens, MLNs, or Peyer’s patches of CD11c-*Ifnar1* ^-/-^ mice and controls eight days after infection with CW3. (**A**) Total CD8 T cells were analyzed for expression of IFNAR1. (**B**) The number or percentage of CW3-tetramer positive CD8 T cells was determined by flow cytometry from the indicated tissue. (**C**) Splenocytes were stimulated *ex vivo* with CW3 peptide and stained for cell surface CD107a and intracellular IFNγ and TNFα. (**D**) RNA extracted from spleens was used to quantitate transcripts for *Ifng*, *Tnfa*, and *Gzmb*. (**E**) The percentage of cells in (C) positive for one, two, or three of the markers of activation is shown. Data in (B) is combined from three experiments and data in (A), (C), (D) and (E) is combined from two experiments with seven mice per data point in (E). Statistical significance was determined by unpaired t test (B, C and D) or 2-way ANOVA (A and C). n.s = p>0.05, * = p≤0.05, ** = p≤0.01, *** = p≤0.001, **** = p≤0.0001.

Similar to NK cells, T cells can express low levels of CD11c when activated. Therefore, we stained T cells for expression of IFNAR1 to determine if it was altered in CD11c-*Ifnar1* ^-/-^ mice. T cells from CD11c-*Ifnar1* ^-/-^ mice had 25% less IFNAR1 in uninfected mice and 50% less IFNAR1 expression 8 days post-inoculation with CW3 (Fig 6A). Thus, expression of IFNAR1 is modestly reduced on T cells in CD11c-*Ifnar1* ^-/-^ mice and may impact their activation in response to virally-induced IFN. Prior work identified the immunodominant CD8 T cell epitope for CW3 [21], which we used to assess the cellular immune response in CD11c-*Ifnar1* ^-/-^ mice. At the peak of T cell expansion eight days after inoculation, we observed an increased proportion of CW3-specific CD8 T cells in spleens (1.4-fold), MLNs (3-fold), and Peyer’s patches (2-fold) of CD11c-*Ifnar1* ^-/-^ mice compared to cre-negative littermate controls (Fig 6B). Likewise, a greater proportion of CD8 T cells in spleens of CD11c-*Ifnar1* ^-/-^ mice responded by expressing IFNγ (1.7-fold), TNFα (1.3-fold), or the degranulation marker CD107a (1.3-fold) following peptide restimulation *ex vivo* (Fig 6C). Among cells that responded to peptide restimulation *in vitro*, there was no significant difference in number of simultaneous functions (IFNγ production, TNFα production, or degranulation) per cell (Fig 6E). To determine if there were increased T cell cytokine responses *in vivo* we quantified transcripts for IFNγ, TNFα, and Granzyme B from spleens of mice 8 days after inoculation with CW3. There were increased transcripts for IFNγ and Granzyme B, while TNFα transcripts were unchanged (Fig 6D). Together, these data indicate that, although there is a decrease in T cell IFNAR1 expression, persistence of CW3 in CD11c-*Ifnar* ^*-/-*^ mice is not correlated with a defect in CD8 T cell expansion or functionality. In fact, there was an increased expansion of equally functional CD8 T cells in CD11c-*Ifnar1* ^-/-^ mice.

### CW3-specific CD8 T cells are functional during persistent infection

**Fig 7:**
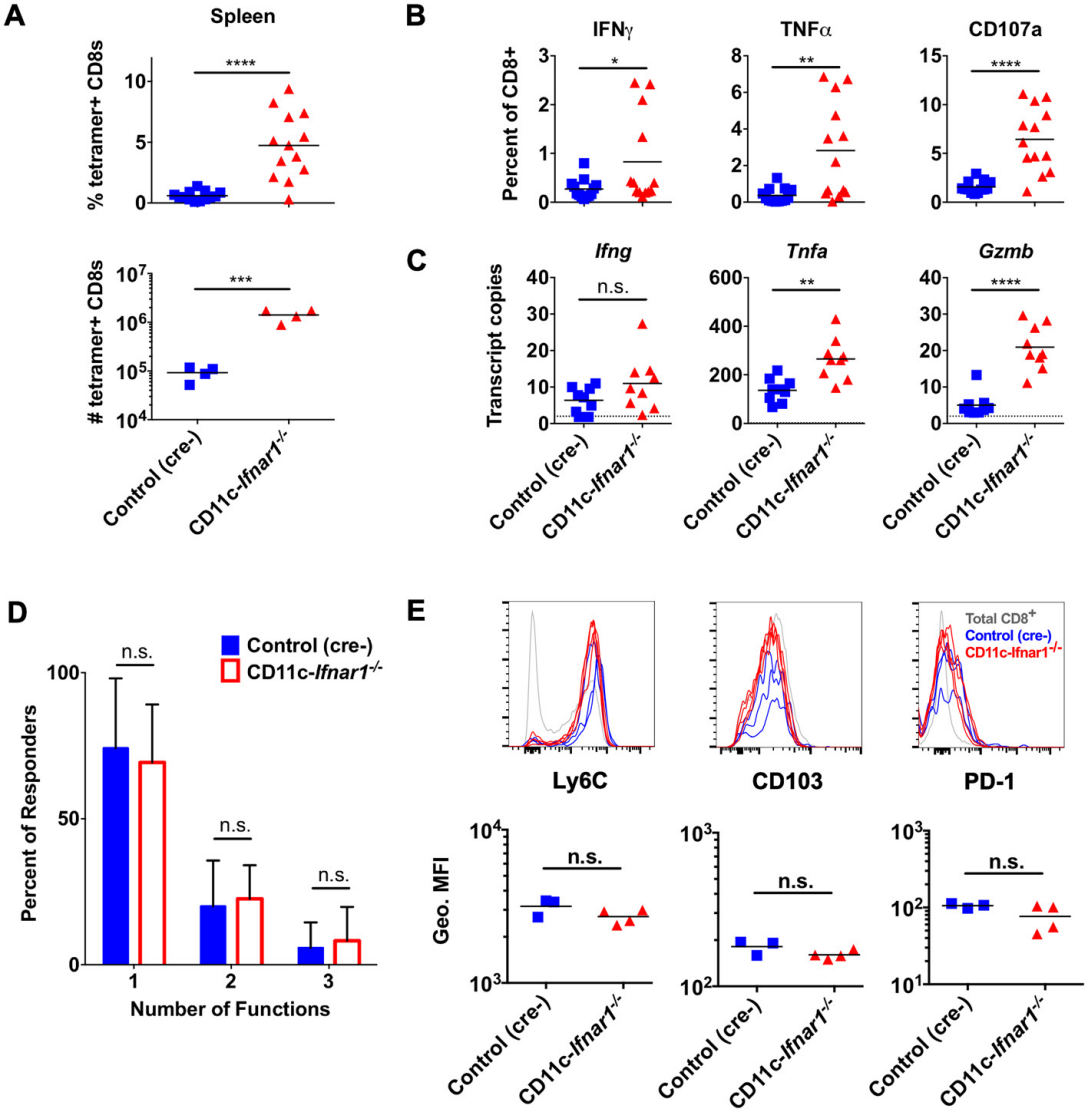
CW3-specific CD8 T cells are functional during persistent infection. Cells were collected from spleens of CD11c-*Ifnar1* ^-/-^ mice and littermate controls 21 days after infection with CW3. The number and percentage of CD8 T cells that were tetramer positive (**A**) or responded to *ex vivo* peptide stimulation (**B**) were determined as in Fig 4. (**C**) RNA extracted from spleens was used to quantitate transcripts for *Ifng*, *Tnfa*, and *Gzmb*. (**D**) The percentage of cells in (B) positive for one, two, or three of the markers of activation is shown. (**E**) Tetramer positive CD8 T cells in the spleens of CD11c-*Ifnar1* ^-/-^ (red lines) and littermate controls (blue lines) were analyzed for cell surface expression of Ly6C, CD103, and PD-1. Grey lines are histograms of total CD8^+^ T cells. Dot plots show the geometric MFI for each marker. Data in (E) is representative of two independent experiments. Data in (A-D) is combined from two (C) or three (A, B, D) experiments with individual mice shown in (A-C) and 13 mice per data point in (D). Statistical significance was determined by unpaired t test (A, B, C) or 2-way ANOVA (D). n.s = p>0.05, * = p≤0.05, ** = p≤0.01, *** = p≤0.001, **** = p≤0.0001.

Early CD8 T cell expansion in persistent infection can be followed by clonal loss or clonal dysfunction [5]. Therefore, we assessed the CW3-specific CD8 T cell response at day 21 after inoculation to determine whether there was a loss of CD8 T cells or a decrease in CD8 T cell functionality. At day 21 there was an increased proportion (8-fold) of CD8 T cells in spleens of CD11c-*Ifnar1* ^-/-^ mice compared to control mice (Fig 7A). There were also an increased proportion of CD8 T cells from spleens of CD11c-*Ifnar1* ^-/-^ mice expressing IFNγ (2.7-fold), TNFα (7.5-fold), or CD107a (4.1-fold) following peptide restimulation *ex vivo* (Fig 7B). Furthermore, there was no difference in the number of simultaneous functions per cell following peptide restimulation (Fig 7D). We also analyzed cell surface expression of the following proteins: 1. PD-1, which is upregulated transiently after activation and sustained on exhausted T cells; 2. Ly6C, which is upregulated and sustained after activation but downregulated on exhausted T cells; 3. CD103, which is transiently downregulated after activation [21,22]. There was no difference in expression of these cell surface proteins on CW3-specific CD8 T cells from CD11c-*Ifnar1* ^-/-^ mice in comparison to cre-negative littermate controls (Fig 7E). Finally, we quantified *in vivo* transcripts for IFNγ, TNFα, and Granzyme B and observed increased transcripts for TNFα and Granzyme B with no significant difference in IFNγ transcripts (Fig 7C). Together, these data indicate that virus-specific CD8 T cells in CD11c-*Ifnar1* ^-/-^ mice are maintained at higher frequencies and remain functional during persistent CW3 infection.

### The adaptive immune response generated in persistently infected CD11c-*Ifnar1* ^-/-^ is functional in an *Ifnar1*-sufficient environment

**Fig 8:**
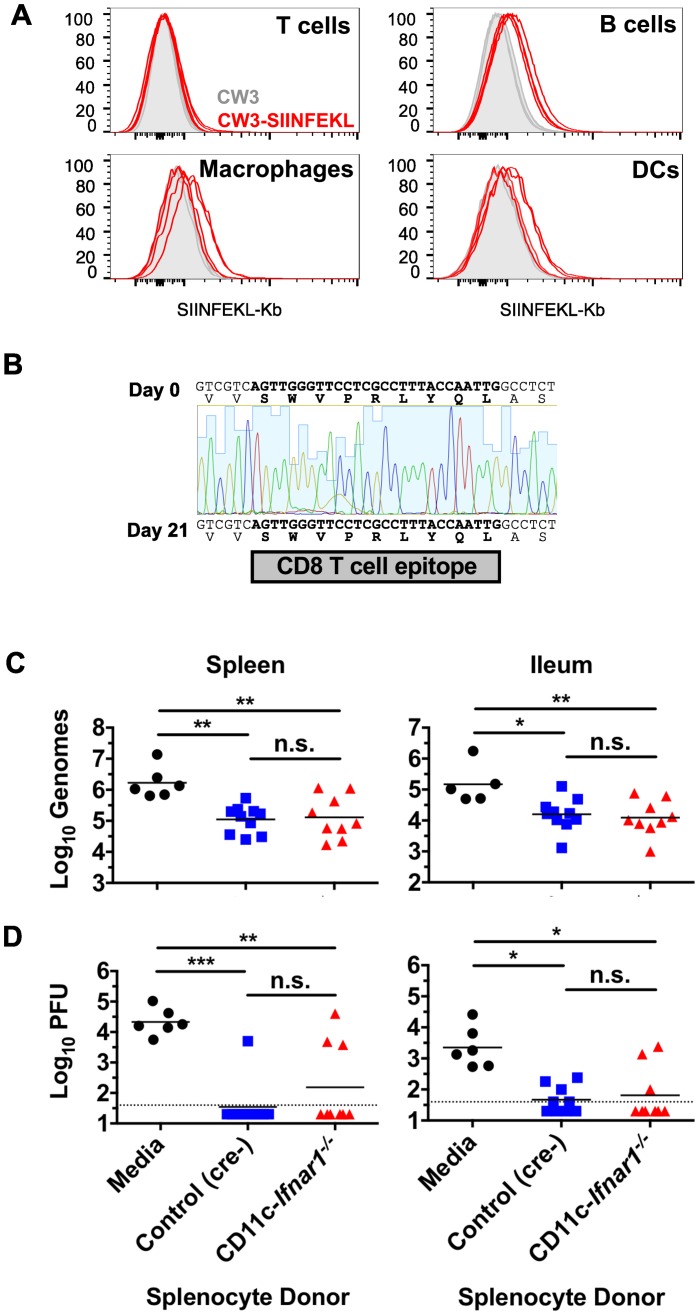
The adaptive immune response generated in persistently infected CD11c-*Ifnar1* ^-/-^ mice is functional in an *Ifnar1*-sufficient environment. (**A**) CD11c-*Ifnar1* ^-/-^ mice were infected with CW3 (filled gray histograms) or CW3 engineered to express the SIINFELK peptide (Red-lined histograms). Three days after inoculation, splenocytes were isolated and stained with an antibody that recognizes H2-K^b^ with SIINFEKL bound in the peptide-binding groove. Overlain histograms from individual mice show fluorescence intensity of staining on T cells (CD3+, CD19-), B cells (CD19+, CD3-), Macrophages (CD3-, CD19-, F4/80+, CD11b^low^), or DCs (CD3-, CD19-, CD11c+, MHCII+) as indicated. (**B**) A portion of the MNoV VP1 gene was amplified by PCR from spleen tissue 21 days after inoculation and sequenced by Sanger sequencing. The shown chromatogram includes the region encoding the immunodominant CD8 T cell epitope and is representative of sequencing results from four mice. (**C and D**) Splenocytes were collected from CD11c-*Ifnar1* ^-/-^ and littermate controls 21 days after infection with CW3. 10^7^ splenocytes were injected I.P. into *Rag* ^*-/-*^ mice that had been infected with CW3 21 days prior. Spleens and Ileums were collected from *Rag* ^*-/-*^ seven days after splenocyte transfer. Viral titers in tissues were determined by qPCR for viral genomes (C) and plaque assay (D). Data in C and D is combined from two experiments with individual mice shown. Statistical significance was determined by one-way ANOVA (A) or Kruskal-Wallis test (B) or. n.s = p>0.05, * = p≤0.05, ** = p≤0.01, *** = p≤0.001, **** = p≤0.0001.

The enhanced adaptive immune response in CD11c-*Ifnar1* ^-/-^ mice (Figs 5–7) correlated with increased early viral replication and reduced viral titers over time, but did not result in elimination of the virus from systemic tissues (Fig 2A). Although DCs are activated (Fig 4) and a CD8 T cell response is initiated (Fig 6), we considered the possibility that virally-infected *Ifnar1*-deficient DCs were unable to present viral antigens to activated T cells. To test this possibility, we utilized a CW3 strain that was engineered to express the peptide SIINFEKL from chicken ovalbumin (CW3-SIINFEKL) and an antibody that recognizes SIINFEKL in the groove of the class I MHC molecule H2-K^b^. DCs, Macrophages, and B cells can be infected by CW3 [19,23] whereas T cells have not been shown to support CW3 infection. Staining of CD11c-*Ifnar1* ^-/-^ splenocytes three days after inoculation showed a modest increase in staining intensity for SIINFEKL-K^b^ on B cells, macrophages, and DCs but not T cells following inoculation with CW3-SIINFEKL compared to the corresponding cells from mice inoculated with CW3 (Fig 8A). Therefore, the absence of *Ifnar1* does not prevent display of antigen on cells susceptible to viral infection. However, it remained possible that CW3 developed mutations to evade recognition by CD8 T cells. To determine if mutation of the CD8 T cell epitope occurred during persistent CW3 infection, sequencing of virus from the spleen was performed 21 days after inoculation. Examination of the sequence of persistent CW3 revealed no deviation in the CD8 T cell epitope sequence from the virus in the inoculum (Fig 8B). Therefore, the CD8 T cell response fails to clear CW3 despite persistence of the un-mutated epitope sequence.

The preceding data suggested that despite presentation of antigen and generation of cellular immunity, a functional adaptive immune response could significantly diminish viral infection but could not fully clear it from *Ifnar1*-deficient innate immune cells. To test the function of the adaptive immune system in a setting where all virally infected cells are *Ifnar1*-sufficient, we utilized a previously published *Rag1* ^-/-^ splenocyte transfer in which CD4 T cells and CD8 T cells contribute to clearance of viral infection [24]. CD11c-*Ifnar1* ^-/-^ or littermate control mice were infected with CW3; 21 days later, splenocytes were isolated and transferred into persistently infected *Rag1* ^-/-^ mice. Six days after splenocyte transfer, genomes were detected by qPCR in spleens and ileums from *Rag1* ^-/-^ and viral titers were determined mice by plaque assay. Splenocytes from either CD11c-*Ifnar1* ^-/-^ or littermate control donors were similarly able to clear PFUs from the spleen and ileum (Fig 6A). Likewise, splenocytes from either donor were similarly able to reduce viral genomes in spleen (10-fold) and ileum (10-fold) (Fig 6B). These data demonstrate that the adaptive immune response in CD11c-*Ifnar1* ^-/-^ mice, including both CD4 and CD8 T cells, is functional in an *Ifnar1*-sufficient environment *in vivo* and suggest that increased viral replication in *Ifnar1*-deficient cells is sufficient for establishing persistent viral replication despite a functional adaptive immune response.

### Dendritic cell-intrinsic IFNAR response is required for clearance of CW3 infection

**Fig 9:**
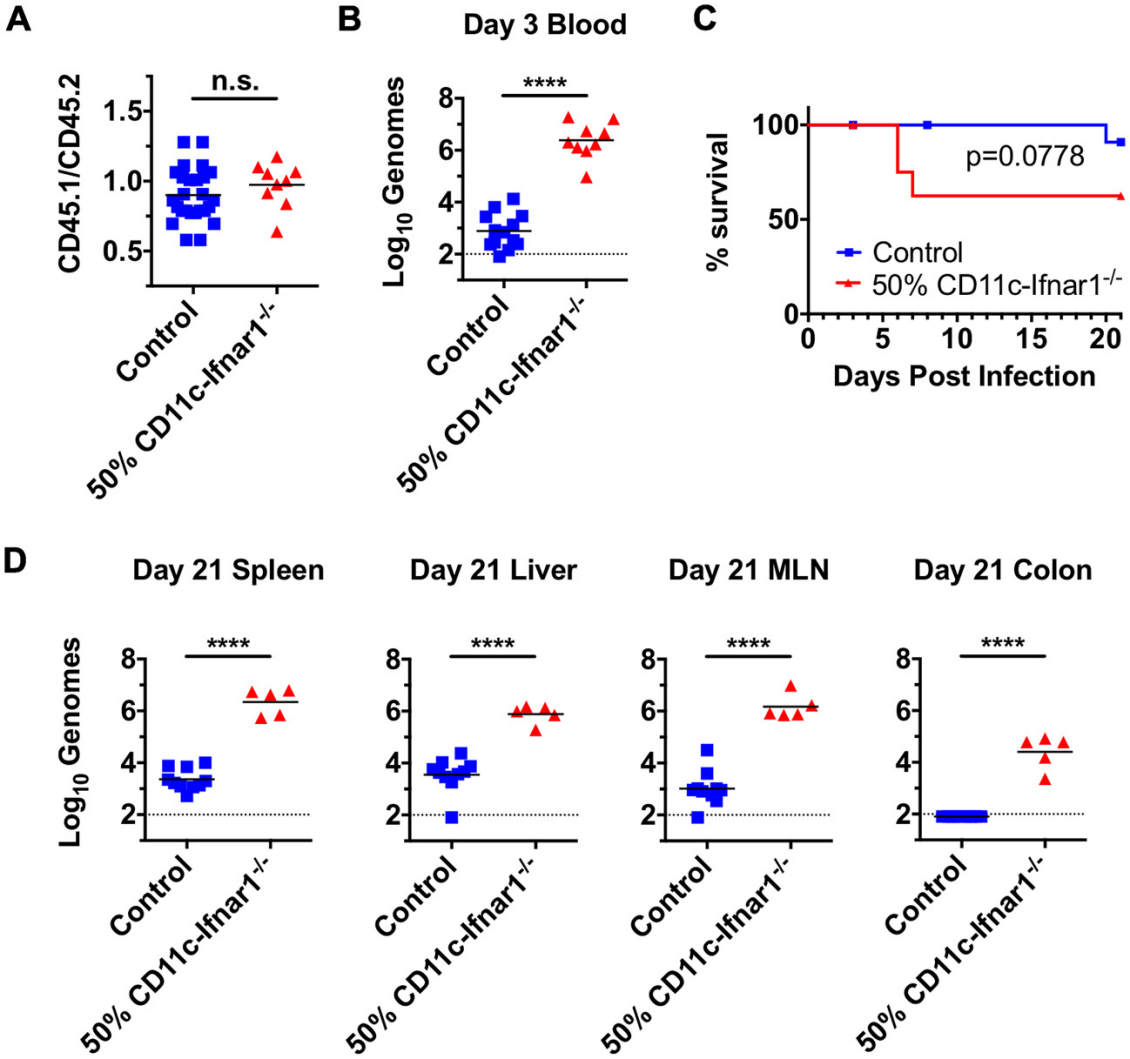
Dendritic cell-intrinsic IFNAR response is required for clearance of CW3 infection. Mixed bone marrow chimeras were generated by reconstitution of irradiated CD45.1 recipients with 50% wild-type CD45.2 bone marrow and 50% wild-type CD45.1 bone marrow (labeled control) or 50% CD11c-*Ifnar1* ^-/-^ (CD45.2) bone marrow and 50% wild-type CD45.1 bone marrow (labeled 50% CD11c-*Ifnar1* ^-/-^). (**A**) Prior to infection, 50% chimerism was confirmed by staining of blood cells with antibodies to CD45.1 and CD45.2. (**B**) Three days after infection with CW3, viral genomes were quantitated per microliter of blood. (**C**) Survival curve of mixed bone marrow chimeras. (**D**) 21 days after infection with CW3, viral genomes were quantitated in spleen, liver, MLN, and colon. Data is combined from two experiments with individual mice shown. Statistical significance was determined by t-test. n.s = p>0.05, **** = p≤0.0001.

The data thus far strongly suggested that DC stimulation of adaptive immunity is not prevented by IFNAR deficiency. We next sought to determine whether the presence of a subpopulation of IFNAR-deficient cells was sufficient for increased CW3 replication. To this end, we generated mixed bone marrow chimeras by reconstitution of irradiated CD45.1 congenic recipients with a 50% mixture of CD11c-*Ifnar1* ^-/-^ bone marrow (CD45.2) and congenic wild-type bone marrow (CD45.1). As a control, a separate cohort of irradiated CD45.1 recipients was reconstituted with a 50% mixture of wild-type bone marrow (CD45.2) and congenic wild-type bone marrow (CD45.1). Six weeks after reconstitution there was an equivalent proportion of CD45.1 and CD45.2 expression on cells in the blood from each group, indicating an equal engraftment of cells from each donor (Fig 9A).

Six weeks after reconstitution, mixed bone marrow chimeras were inoculated with CW3. Three days after inoculation, the amount of viral genomes in the blood was determined as a measure of early systemic viral replication. 50% CD11c-*Ifnar1* ^-/-^ bone marrow chimeras were unable to control early systemic viral replication with 3,000-fold more viral genomes per microliter of blood compared to control chimeras (Fig 9B). Furthermore, early shedding of viral genomes in the feces of 50% CD11c-*Ifnar1* ^-/-^ bone marrow chimeras on days five and seven was increased 37-fold and 10-fold respectively compared to control chimeras (S2A Fig). This demonstrates that the presence of a subpopulation of IFNAR-deficient DCs is sufficient for increased early viral replication. Correspondingly, the presence of mixed wild-type leukocytes is not sufficient to enable early viral control.

Unexpectedly, mixed bone marrow chimeras had increased susceptibility to infection; three out of eight 50% CD11c-*Ifnar1* ^-/-^ chimeras died prior to day eight. Additionally, one control mouse died on day 20 (Fig 9C). On day 21, we observed reduced numbers of lymphocytes in spleens of surviving 50% CD11c-*Ifnar1* ^-/-^ compared to control chimeras (S2B Fig). The increased lethality and reduced number of leukocytes suggested that the process of bone marrow transplantation results in an immunocompromised state and increased susceptibility to CW3. However, the number and chimerism of DCs in 50% CD11c-*Ifnar1* ^-/-^ bone marrow chimeras was not significantly different from that control chimeras (S2C and S2D Fig) allowing us to compare viral titers in surviving mice from these two groups. Viral genomes were significantly higher in the spleen (1,000-fold), liver (200-fold), MLN (1,400-fold), and colon (>300-fold) of 50% CD11c-*Ifnar1* ^-/-^ bone marrow chimeras compared to control chimeras (Fig 9D). These data indicate that the presence of mixed wild-type DCs does not rescue control of persistent viral replication. Taken together with the preceding data, this suggests that the presence of a subpopulation of cells with increased susceptibility to CW3 infection is sufficient for increased early and persistent viral replication.

## Discussion

We have shown here that selective deficiency in the type I IFN response in DCs results in MNoV persistence. Prior studies utilizing the LCMV model causally link diminished CD8 T cell functionality to viral persistence [6] and other studies have demonstrate a role for type I IFN in regulating this process [25–29]. Furthermore, antigen abundance has been positively correlated with the degree of CD8 T cell dysfunction [5,30]. The MNoV persistence demonstrated herein contrasts with this paradigm as it was not associated with failure to generate an adaptive immune response or loss of adaptive immune function. In fact, there is an enhanced adaptive immune response commensurate with increased viral replication, and this enhanced adaptive immune response is sustained during persistent infection. These data demonstrate that an innate immune defect in type I IFN can result in viral persistence without attendant effects on the adaptive arm of the immune system. These results provide a different view of viral persistence than prior work in the LCMV model. However, it is important to note that antigen abundance may be lower in the MNoV model relative to the LCMV model. Additionally, the importance of type I IFN in protecting against lethal infection differs between CW3 and LCMV [17,25], providing a potential basis for differing roles of type I IFN in these two models.

In the MNoV model of persistence studied here, we observed that increased viral replication due to defects in the type I IFN pathway is linked to viral persistence: *Ifnar* ^*-/-*^ mice have high early viral replication and succumb to infection; *Irf3x7* ^*-/-*^ mice have marginally lower peak viral replication [17] and allow CW3 to persist; lineage specific deletion of *Ifnar1* in CD11c or LysM expressing cells also results in less viral replication than *Ifnar* ^*-/-*^ [17] but allows MNoV persistence; *Ifih1* ^*-/-*^ mice have small increases in viral replication [31] but are able to clear the infection; lineage specific deletion of *Ifnar1* in Villin-expressing cells leads to no change in viral titer and clearance. The relatively greater effect of IFNAR in myeloid cells rather than intestinal epithelial cells is likely due to the preferential replication of CW3 in myeloid cells and/or low IFNAR expression on intestinal epithelial cells [32]. Together, these findings support a model where varying levels of type I IFN signaling inversely correlate with peak viral replication and viral clearance.

**Fig 10:**
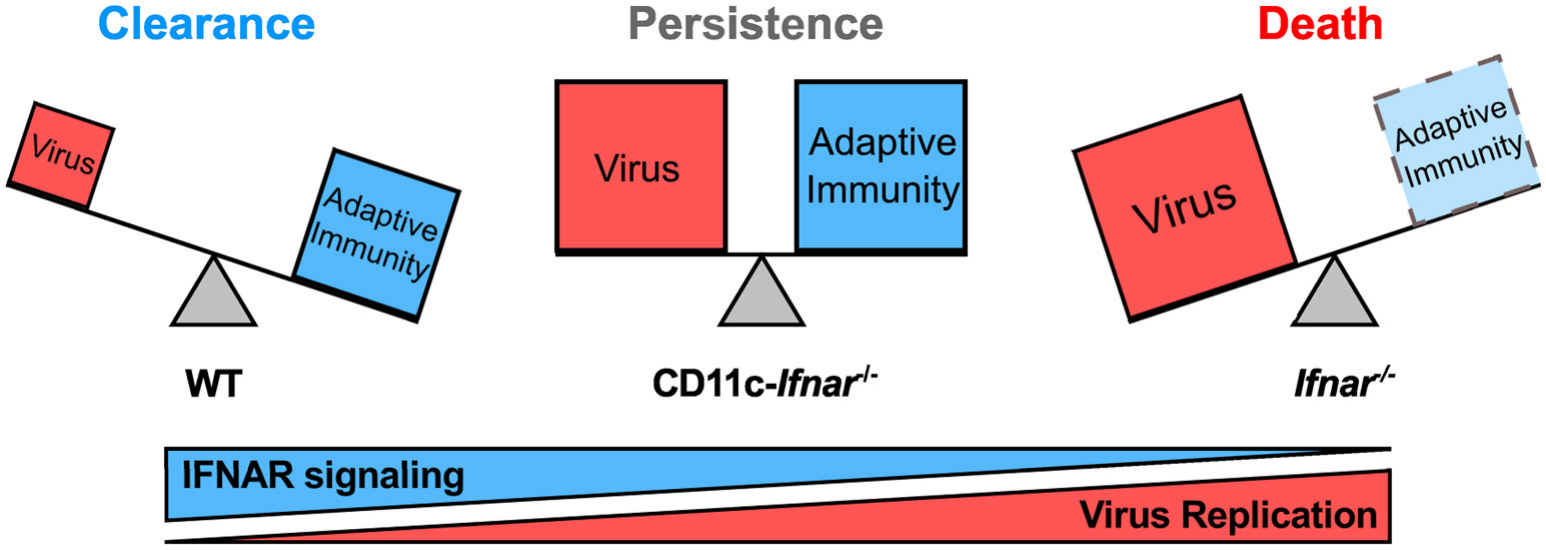
Model for the relationship between IFNAR signaling, viral replication, the adaptive immune response, and persistence. The contribution of the adaptive immune response depends on the level of type I IFN deficiency. When the type I IFN response is intact, innate immunity is relatively effective at control of MNoV and a small magnitude of adaptive immune response is sufficient to clear the infection. When a susceptible cell type (i.e. DCs) lacks the ability to respond to type I IFN, MNoV replication is increased, a larger magnitude of adaptive immune response is elicited, and infection is controlled but not cleared. When type I IFN signaling is completely absent, MNoV replication is at a maximum and the host succumbs to infection prior to generation of a protective adaptive immune response.

An additional conclusion from these data is that viral titer is not always positively correlated with adaptive immune dysfunction. In the LCMV model, increased viral titer and associated antigen levels are predictive of T cell clonal deletion or loss of T cell function [5]. Unlike the LCMV model, persistent infection of CW3 in CD11c-*Ifnar1* ^-/-^ mice is not correlated with loss of CD8 T cell numbers or function. Instead, increased viral titers are balanced by a commensurate increase in CD8 T cell expansion and antibody production: wild type mice have intact innate control and a relatively lower level of CD8 T cell expansion and antibody production; *Ifnar1* ^-/-^ mice represent an unbalanced scenario with uncontrolled early viral replication resulting in death prior to development of an adaptive response; CD11c-*Ifnar1* ^-/-^ mice represent a ‘sweet spot’ where increased viral growth is controlled but not cleared by an enhanced adaptive immune response resulting a persistent balance between viral replication and clearance (Fig 10). This model is simplistic and does not consider other components of the innate immune response, but represents a rationale for viral persistence in the presence of an enhanced adaptive immune response.

The implication of this model is that the primary mechanistic basis for certain persistent infections could be manipulation of IFN, or other innate antiviral responses. Likewise, these data imply that some persistent infections could be treated by modulating innate immunity, particularly type I IFN, rather than targeting adaptive immune responses. Indeed, both of these principles are manifest in persistent HCV infection in humans. The HCV NS3/4A protease inhibits the induction of IFN by cleaving MAVS [33]. Prior to the recent development of small molecule inhibitors of HCV replication, the most effective therapy for persistent HCV infection included IFN treatment. So, successful treatment for HCV serves to circumvent the viral inhibition of IFN induction. Thus, HCV may be an example of a medically relevant persistent viral infection that persists due, in part, to loss of innate immune function. Persistence of other continuously replicating RNA viruses, such as chikungunya, measles, polyomavirus, may be similarly due to ineffective innate responses.

## Materials and Methods

### Mice and infections

*Ifnar1* ^*-/-*^ (Ifnar1^tm1Agt^), *Ifnar1* ^*flox/flox*^ (Ifnar1^tm1Uka^), LysM-cre (Lyz2^tm1(cre)Ifo^), CD11c-cre (Tg(Itgax-cre,-EGFP)), villin-cre (Tg(Vil1-cre)Gum), *IRF3* ^*-/-*^ (Irf3^tm1Ttg^), *IRF7* ^*-/-*^ (Irf7^tm1Ttg^), *Ifih1* ^*-/-*^, *Mavs* ^*-/-*^, *Ticam1* ^*-/-*^, and *Myd88* ^*-/-*^ mice were maintained at Washington University as described [34]. All mice were bred on C57BL/6 background. Cre-negative littermates from *Ifnar1* ^*flox/flox*^ x villin-cre, *Ifnar1* ^*flox/flox*^ x LysM-cre, and *Ifnar1* ^*flox/flox*^ x CD11c-cre crosses were used as controls. In few experiments as indicated, C57BL/6 mice were used as controls. Mice were infected at 7–12 weeks of age. MNoV strain CW3 stocks were generated as described [13] and diluted in media to a concentration of 4x10^7^ PFU per mL. 10^6^ PFU (25 μL) was administered orally by pipet to each mouse. CW3-SIINFEKL was generated by inserting the epitope encoding sequence into the ORF1 polyprotein between NS4 and NS5 as previously described [35].

CD45.1 congenic mice used as bone marrow recipients were obtained from Jackson Laboratories (Bar Harbor, ME) and housed at Oregon Health & Science University. Bone marrow was isolated from CD45.1 congenic mice and CD45.2 experimental mice and mixed at a 1:1 ratio. Recipient CD45.1 mice were irradiated with 1200rad and 2.5x10^6^ total donor bone marrow cells were injected intravenously. Mice were given ciprofloxacin in the drinking water for two weeks and used for experiments after six weeks.

### Ethics statement

Protocols for animal care and use were approved by the Animal Studies Committee at Washington University in St. Louis (protocol #20140244) and Oregon Health & Science University (protocol # IP00000228) according to standards set forth in the *Animal Welfare Act*.

### Quantitative RT-PCR

Tissues were dissected and flash frozen. RNA from tissues was isolated using Trizol (Life Technologies) and DNA was removed using the DNAfree kit (Life Technologies). RNA from stool was isolated using ZR Viral RNA kit (Zymoresearch). RNA was reverse transcribed using ImPromII reverse transcriptase system (Promega). MNoV genome quantities and transcripts for the housekeeping gene, ribosomal protein S29 (RPS29) were detected using the following primers and probes: MNoV Forward primer, CACGCCACCGATCTGTTCTG; MNoV MGB Probe, CGCTTTGGAACAATG; MNoV Reverse primer, GCGCTGCGCCATCACTC; RPS29 Forward primer, GCAAATACGGGCTGAACATG; RPS29 Probe, CCTTCGCGTACTGCCGGAAGC; RPS29 Reverse primer, GTCCAACTTAATGAAGCCTATGTC. Transcripts for *Ifit1*(Mm.PT.58.32674307), *Isg15*(Mm.PT.58.41476392.g), *Cxcl9*(Mm.PT.58.13098261), *Ccl5*(Mm.PT.58.43548565), *Ifng*(Mm.PT.58.30096391), *Tnfa*(Mm.PT.58.12575861), *Gzmb* (Mm.PT.58.12967220) were detected using PrimeTime qPCR assays from integrated DNA technologies (IDT). Copy number was determined using a standard curve generated by dilution of a plasmid encoding the target sequence of interest.

### ELISA

Immulon 2HB plates (Thermo Fisher) were coated with UV inactivated MNoV virions or bacterially-produced MNoV NS1 (amino acids 28–114) in PBS. Plates were washed three times in ELISA wash buffer (0.15M NaCl, 0.05% Tween 20) and blocked with 3% bovine serum albumin. Serum was diluted in ELISA III buffer (0.15M NaCl, 0.001M EDTA, 0.05M Tris, 0.05% Tween 20, 0.1% BSA, pH 7.4) and added to plate for 1 hour at 37°C. Unbound serum antibody was removed by washing four times in ELISA wash buffer. Goat-anti-mouse-HRP (Jackson Immunoresearch) was diluted 1:1000 in ELISA III buffer and added to plate for 1 hour at 37°C. Plate was washed four times in ELISA wash buffer. HRP activity was detected by adding ELISA substrate buffer (0.1M sodium citrate, 1mM ABTS, 0.016% hydrogen peroxide) and stopping in 0.2N Phosphoric Acid. Absorbance was measured at 415nm.

### Serum neutralization assay

Serum from mice infected for 35 days with MNoV was heat inactivated at 55°C for 30 minutes and diluted 10-fold in cell culture media (DMEM supplemented with 10% fetal calf serum, L-Glutamine, HEPES, and Pennecilin/Streptamycin). 10 PFU of MNoV strain CW3 was added to each serum dilution in a total volume of 100 μL and incubated at room temperature for 30 minutes prior to inoculation of RAW cell monolayers in six-well plates. Inoculation of RAW cells and plaque assay were performed as described [13].

### Cell isolation and flow cytometry

Spleens, mesenteric lymph nodes, and Peyer’s patches were removed and forced through 70 μM cell strainers to create single cell suspensions. Red blood cells in splenocyte suspensions were lysed with ammonium chloride potassium (ACK) lysing buffer (Sigma). Dead cells were stained with live/dead Aqua (Life Technologies). Surface antigens were stained with the following antibodies: CD11c (eBioscience, clone N418), MHCII (BioLegend, clone M5/114.15.2), CD3ε (BioLegend, clone 145-2C11), CD19 (BD biosciences, clone 1D3), CD8α (BD biosciences, clone 53–6.7), CD4 (BioLegend, clone RM4-5), CD40, CD80, CD86, MHCI-K^b^ (BD biosciences, clone AF6-88.5), CD44 (BD biosciences, clone IM7), PD-1 (BioLegend, clone RMP1-14), CD103 (BioLegend, clone 2E7), Ly6C (BioLegend, clone HK1.4), NKp46 (BioLegend, clone 29A1.4), IFNAR1 (BioLegend, clone MAR1-5A3), KLRG1 (Tonbo, clone 2F1), CD62L (BioLegend, clone MEF-14), SIINFEKL peptide in grove antibody (BioLegend, clone 25-D1.16), CD45.1 (BioLegend clone A20), CD45.2 (BioLegend clone 104). MHCI K^b^ tetramers with the CW3 immunodominant epitope SWVPRLYQL were generated as described [21].

### Peptide restimulation and intracellular cytokine staining

5x10^6^ splenocytes were incubated in 96-well round bottom plates in triplicate with Monensin (BioLegend), Brefeldin-A (BioLegend), CW3 peptide SWVPRLYQL (0.4 μg/mL), and the CD107a antibody (eBioscience, clone ABL-93) for four to five hours at 37°C. Cells were surface stained followed by fixation and permeabilization using the cytofix/cytoperm kit (BD biosciences). Permeabilized cells were stained with antibodies for TNFα (BioLegend, clone MP6-XT22), IFNγ (BD biosciences, clone XMG1.2) and analyzed by flow cytometry.

### Splenocyte adoptive transfer assay

Adoptive transfer of splenocytes into persistently infected *Rag1* ^*-/-*^ mice was performed as described [24]. Briefly, splenocytes were isolated from donors that had been infected with CW3 and red blood cells were lysed. 10^7^ splenocytes were injected i.p. into *Rag1* ^*-/-*^ recipients that had been infected with CW3 for 21 days. Six days after transfer, tissues were harvested. PFU were determined by plaque assay and genomes were quantified by qPCR.

## Supporting Information

S1 Fig NK cells have reduced IFNAR1 expression in CD11c-*Ifnar1* ^-/-^ mice and similar expression of other phenotypic markers. NK cells (CD3-negative, CD19-negative, NKp46-positive) from CD11c-*Ifnar1* ^-/-^ mice and C57BL/6 controls 3 days after innoculation with CW3 were quantified (**A**) and stained for surface markers (**B**). The geometric mean fluorescence intensity (Geo. MFI) is shown for CD11c, IFNAR1, and markers of activation: KLRG1, CD62L, and Ly6C. Data is combined from two experiments. Statistical significance was determined by unpaired t test. n.s = p>0.05, * = p≤0.05, **** = p≤0.0001.
(TIF)

S2 Fig CD11c-*Ifnar1* ^-/-^ mixed bone marrow chimeras have similar DC numbers and chimerism 21 days after CW3 infection. (**A**) Feces were collected from the mixed bone marrow chimeras in Fig 9 on the indicated days and viral genomes were quantified. (**B-D**) 21 days after inoculation with CW3, splenocytes were isolated from mixed bone marrow chimeras in Fig 9. (**B**) Numbers of total splenocytes, CD3+ T cells, CD19+ B cells, and CD11c+ MHCII+ DCs per spleen. (**C**) Percentage of each indicated cell subset. (**D**) Ratio of CD45.1 (wild-type donor and recipient) to CD45.2 (CD11c-*Ifnar1* ^-/-^ donor or wild-type control donor) cells of the indicated cell subset. Data is combined from two experiments. Statistical significance was determined by two-way ANOVA (A) or t-test (B-D). n.s = p>0.05, * = p≤0.05, ** = p≤0.01, *** = p≤0.001, **** = p≤0.0001.
(TIF)

## References

[1] Ng CT, Sullivan BM, Oldstone MB. The role of dendritic cells in viral persistence. Curr Opin Virol. 2011;1: 160–166. 10.1016/j.coviro.2011.05.006 21909344 PMC3167161

[2] Shin H, Wherry EJ. CD8 T cell dysfunction during chronic viral infection. Curr Opin Immunol. 2007;19: 408–415. 10.1016/j.coi.2007.06.004 17656078

[3] Virgin HW, Wherry EJ, Ahmed R. Redefining Chronic Viral Infection. Cell. 2009;138: 30–50. 10.1016/j.cell.2009.06.036 19596234

[4] Bergmann CC, Lane TE, Stohlman SA. Coronavirus infection of the central nervous system: host–virus stand-off. Nat Rev Microbiol. 2006;4: 121–132. 10.1038/nrmicro1343 16415928 PMC7096820

[5] Wherry EJ, Blattman JN, Murali-Krishna K, van der Most R, Ahmed R. Viral persistence alters CD8 T-cell immunodominance and tissue distribution and results in distinct stages of functional impairment. J Virol. 2003;77: 4911–27. 12663797 10.1128/JVI.77.8.4911-4927.2003 PMC152117

[6] Barber DL, Wherry EJ, Masopust D, Zhu B, Allison JP, Sharpe AH, et al Restoring function in exhausted CD8 T cells during chronic viral infection. Nature. 2006;439: 682–687. 10.1038/nature04444 16382236

[7] Bergmann CC, Altman JD, Hinton D, Stohlman SA. Inverted immunodominance and impaired cytolytic function of CD8+ T cells during viral persistence in the central nervous system. J Immunol. 1999;163: 3379–87. 10477608

[8] Ramakrishna C, Stohlman SA, Atkinson RA, Hinton DR, Bergmann CC. Differential Regulation of Primary and Secondary CD8+ T Cells in the Central Nervous System. J Immunol. 2004;173: 6265–6273. 10.4049/jimmunol.173.10.6265 15528365

[9] Hsu CC, Riley LK, Wills HM, Livingston RS. Persistent infection with and serologic cross-reactivity of three novel murine noroviruses. Comp Med. 2006;56: 247–51. 16941951

[10] Thackray LB, Wobus CE, Chachu KA, Liu B, Alegre ER, Henderson KS, et al Murine Noroviruses Comprising a Single Genogroup Exhibit Biological Diversity despite Limited Sequence Divergence. J Virol. 2007;81: 10460–10473. 10.1128/JVI.00783-07 17652401 PMC2045448

[11] Saito M, Goel-Apaza S, Espetia S, Velasquez D, Cabrera L, Loli S, et al Multiple Norovirus Infections in a Birth Cohort in a Peruvian Periurban Community. Clin Infect Dis. 2014;58: 483–491. 10.1093/cid/cit763 24300042 PMC3905757

[12] Atmar RL, Opekun AR, Gilger MA, Estes MK, Crawford SE, Neill FH, et al Norwalk Virus Shedding after Experimental Human Infection. Emerg Infect Dis. 2008;14: 1553–1557. 10.3201/eid1410.080117 18826818 PMC2609865

[13] Nice TJ, Strong DW, McCune BT, Pohl CS, Virgin HW. A Single-Amino-Acid Change in Murine Norovirus NS1/2 Is Sufficient for Colonic Tropism and Persistence. J Virol. 2013;87: 327–334. 10.1128/JVI.01864-12 23077309 PMC3536416

[14] Baldridge MT, Nice TJ, McCune BT, Yokoyama CC, Kambal A, Wheadon M, et al Commensal microbes and interferon-λ determine persistence of enteric murine norovirus infection. Science (80-). 2015;347: 266–269. 10.1126/science.1258025 PMC4409937 25431490

[15] Nice TJ, Baldridge MT, McCune BT, Norman JM, Lazear HM, Artyomov M, et al Interferon-λ cures persistent murine norovirus infection in the absence of adaptive immunity. Science (80-). 2015;347: 269–273. 10.1126/science.1258100 PMC4398891 25431489

[16] Lazear HM, Nice TJ, Diamond MS. Interferon-λ: Immune Functions at Barrier Surfaces and Beyond. Immunity. Elsevier Inc.; 2015;43: 15–28. 10.1016/j.immuni.2015.07.001 PMC4527169 26200010

[17] Thackray LB, Duan E, Lazear HM, Kambal A, Schreiber RD, Diamond MS, et al Critical Role for Interferon Regulatory Factor 3 (IRF-3) and IRF-7 in Type I Interferon-Mediated Control of Murine Norovirus Replication. J Virol. 2012;86: 13515–13523. 10.1128/JVI.01824-12 23035219 PMC3503103

[18] Karst SM, Wobus CE, Lay M, Davidson J, Virgin HW. STAT1-dependent innate immunity to a Norwalk-like virus. Science (80-). 2003;299: 1575–8. 10.1126/science.1077905 12624267

[19] Wobus CE, Karst SM, Thackray LB, Chang K-O, Sosnovtsev S V., Belliot G, et al Replication of Norovirus in Cell Culture Reveals a Tropism for Dendritic Cells and Macrophages. Michael Emerman, editor. PLoS Biol. 2004;2: e432 10.1371/journal.pbio.0020432 15562321 PMC532393

[20] Bachmann MF, Kopf M. The role of B cells in acute and chronic infections. Curr Opin Immunol. 1999;11: 332–9. 10375561 10.1016/s0952-7915(99)80053-3

[21] Tomov VT, Osborne LC, Dolfi D V, Sonnenberg GF, Monticelli L a, Mansfield K, et al Persistent Enteric Murine Norovirus Infection Is Associated with Functionally Suboptimal Virus-Specific CD8 T Cell Responses. J Virol. 2013;87: 7015–7031. 10.1128/JVI.03389-12 23596300 PMC3676130

[22] Wherry EJ, Ha S-J, Kaech SM, Haining WN, Sarkar S, Kalia V, et al Molecular Signature of CD8+ T Cell Exhaustion during Chronic Viral Infection. Immunity. 2007;27: 824 10.1016/j.immuni.2007.11.006 17950003

[23] Jones MK, Watanabe M, Zhu S, Graves CL, Keyes LR, Grau KR, et al Enteric bacteria promote human and mouse norovirus infection of B cells. Science (80-). 2014;346: 755–759. 10.1126/science.1257147 PMC4401463 25378626

[24] Chachu KA, LoBue AD, Strong DW, Baric RS, Virgin HW. Immune Mechanisms Responsible for Vaccination against and Clearance of Mucosal and Lymphatic Norovirus Infection. Estes MK, editor. PLoS Pathog. 2008;4: e1000236 10.1371/journal.ppat.1000236 19079577 PMC2587711

[25] Ou R, Zhou S, Huang L, Moskophidis D. Critical role for alpha/beta and gamma interferons in persistence of lymphocytic choriomeningitis virus by clonal exhaustion of cytotoxic T cells. J Virol. 2001;75: 8407–23. 11507186 10.1128/JVI.75.18.8407-8423.2001 PMC115086

[26] Wilson EB, Yamada DH, Elsaesser H, Herskovitz J, Deng J, Cheng G, et al Blockade of Chronic Type I Interferon Signaling to Control Persistent LCMV Infection. Science (80-). 2013;340: 202–207. 10.1126/science.1235208 PMC3704950 23580528

[27] Teijaro JR, Ng C, Lee AM, Sullivan BM, Sheehan KCF, Welch M, et al Persistent LCMV Infection Is Controlled by Blockade of Type I Interferon Signaling. Science (80-). 2013;340: 207–211. 10.1126/science.1235214 PMC3640797 23580529

[28] Shaabani N, Khairnar V, Duhan V, Zhou F, Tur RF, Häussinger D, et al Two separate mechanisms of enforced viral replication balance innate and adaptive immune activation. J Autoimmun. 2016;67: 82–9. 10.1016/j.jaut.2015.10.004 26553386

[29] Nakayama Y, Plisch EH, Sullivan J, Thomas C, Czuprynski CJ, Williams BRG, et al Role of PKR and Type I IFNs in Viral Control during Primary and Secondary Infection. Baric RS, editor. PLoS Pathog. 2010;6: e1000966 10.1371/journal.ppat.1000966 20585572 PMC2891951

[30] Richter K, Brocker T, Oxenius A. Antigen amount dictates CD8+T-cell exhaustion during chronic viral infection irrespective of the type of antigen presenting cell. Eur J Immunol. 2012;42: 2290–2304. 10.1002/eji.201142275 22653665

[31] McCartney SA, Thackray LB, Gitlin L, Gilfillan S, Virgin HW IV, Colonna M. MDA-5 Recognition of a Murine Norovirus. Baric RS, editor. PLoS Pathog. 2008;4: e1000108 10.1371/journal.ppat.1000108 18636103 PMC2443291

[32] Mahlakõiv T, Hernandez P, Gronke K, Diefenbach A, Staeheli P. Leukocyte-Derived IFN-α/β and Epithelial IFN-λ Constitute a Compartmentalized Mucosal Defense System that Restricts Enteric Virus Infections. Greenberg HB, editor. PLOS Pathog. 2015;11: e1004782 10.1371/journal.ppat.1004782 25849543 PMC4388470

[33] Li X-D, Sun L, Seth RB, Pineda G, Chen ZJ. Hepatitis C virus protease NS3/4A cleaves mitochondrial antiviral signaling protein off the mitochondria to evade innate immunity. Proc Natl Acad Sci. 2005;102: 17717–17722. 10.1073/pnas.0508531102 16301520 PMC1308909

[34] Cadwell K, Liu JY, Brown SL, Miyoshi H, Loh J, Lennerz JK, et al A key role for autophagy and the autophagy gene Atg16l1 in mouse and human intestinal Paneth cells. Nature. 2008;456: 259–263. 10.1038/nature07416 18849966 PMC2695978

[35] Osborne LC, Monticelli LA, Nice TJ, Sutherland TE, Siracusa MC, Hepworth MR, et al Virus-helminth coinfection reveals a microbiota-independent mechanism of immunomodulation. Science (80-). 2014;345: 578–582. 10.1126/science.1256942 PMC4548887 25082704

