# Correction: Type I Interferon Receptor Deficiency in Dendritic Cells Facilitates Systemic Murine Norovirus Persistence Despite Enhanced Adaptive Immunity

**DOI:** 10.1371/journal.ppat.1006223

**Published:** 2017-02-17

**Authors:** Timothy J. Nice, Lisa C. Osborne, Vesselin T. Tomov, David Artis, E. John Wherry, Herbert W. Virgin

The following information is missing from the Funding section: TJN was supported by a faculty development award from the Sunlin & Priscilla Chou Foundation
