# Supplementary figures and images for: Type I Interferon Receptor Deficiency in Dendritic Cells Facilitates Systemic Murine Norovirus Persistence Despite Enhanced Adaptive Immunity
Source: PLoS Pathog. 2016 Jun 21;12(6):e1005684. doi: 10.1371/journal.ppat.1005684 (PMC4915689; doi:10.1371/journal.ppat.1005684)

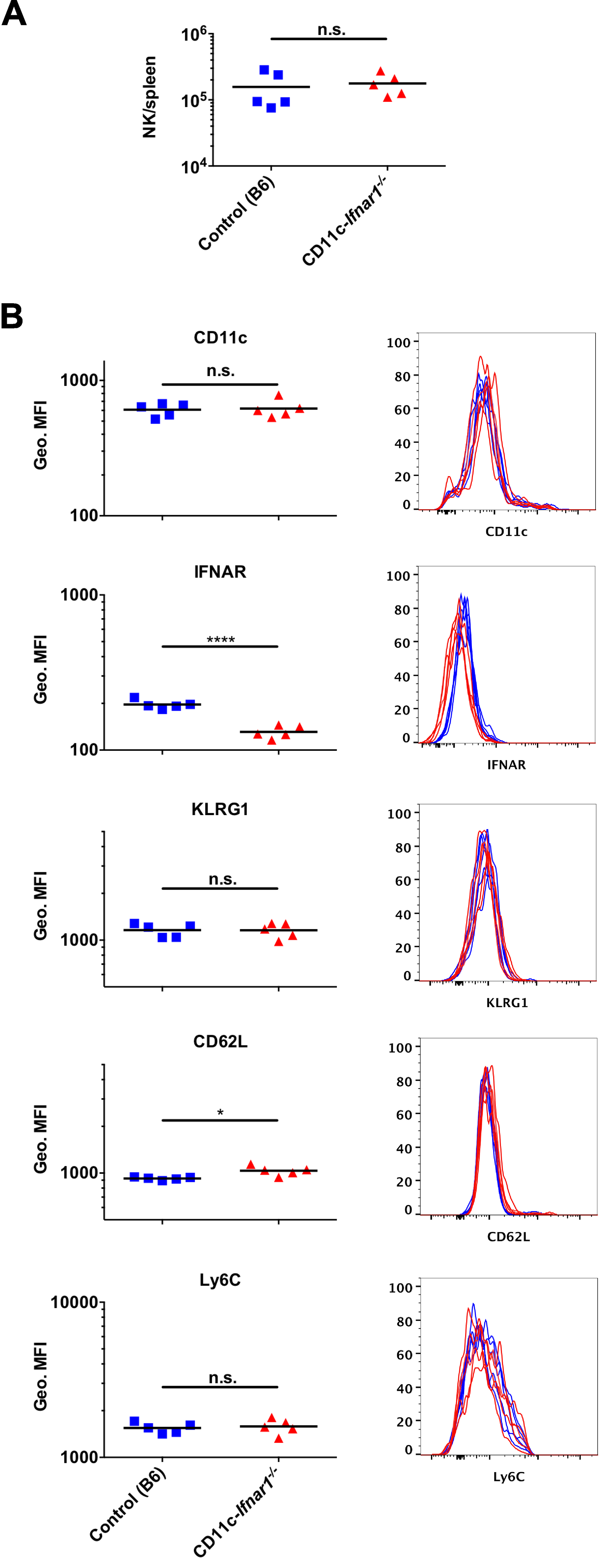

Supplement: S1 Fig — NK cells (CD3-negative, CD19-negative, NKp46-positive) from CD11c-Ifnar1 -/- mice and C57BL/6 controls 3 days after innoculation with CW3 were quantified (A) and stained for surface markers (B). The geometric mean fluorescence intensity (Geo. MFI) is shown for CD11c, IFNAR1, and markers of activation: KLRG1, CD62L, and Ly6C. Data is combined from two experiments. Statistical significance was determined by unpaired t test. n.s = p>0.05, * = p≤0.05, **** = p≤0.0001. (TIF) [file ppat.1005684.s001.tif]

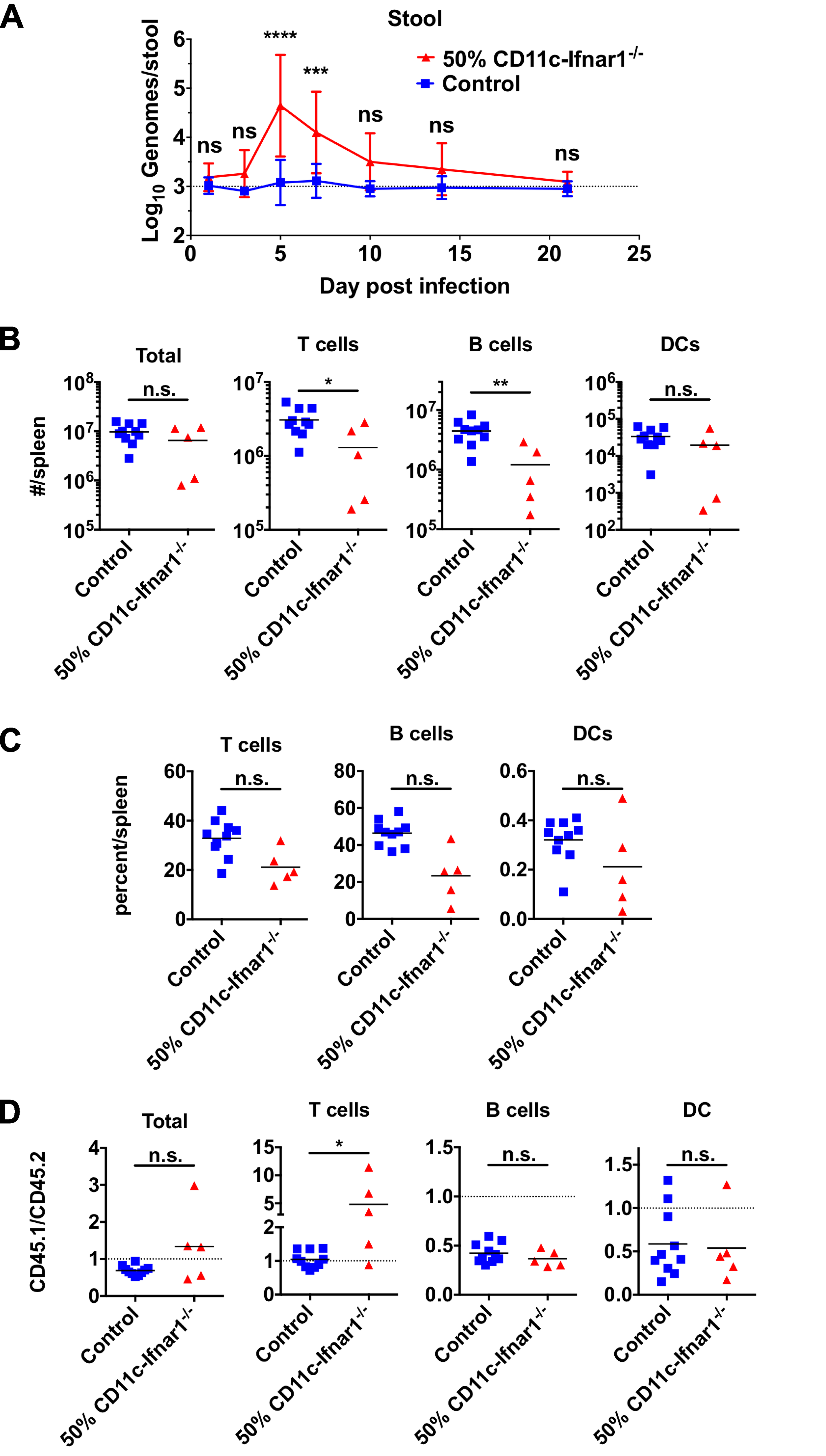

Supplement: S2 Fig — (A) Feces were collected from the mixed bone marrow chimeras in Fig 9 on the indicated days and viral genomes were quantified. (B-D) 21 days after inoculation with CW3, splenocytes were isolated from mixed bone marrow chimeras in Fig 9. (B) Numbers of total splenocytes, CD3+ T cells, CD19+ B cells, and CD11c+ MHCII+ DCs per spleen. (C) Percentage of each indicated cell subset. (D) Ratio of CD45.1 (wild-type donor and recipient) to CD45.2 (CD11c-Ifnar1 -/- donor or wild-type control donor) cells of the indicated cell subset. Data is combined from two experiments. Statistical significance was determined by two-way ANOVA (A) or t-test (B-D). n.s = p>0.05, * = p≤0.05, ** = p≤0.01, *** = p≤0.001, **** = p≤0.0001. (TIF) [file ppat.1005684.s002.tif]
